# Commentary: Percutaneous tracheostomy: comparison of three different methods with respect to tracheal cartilage injury in cadavers—randomized controlled study

**DOI:** 10.3389/pore.2023.1611451

**Published:** 2023-09-08

**Authors:** Eckart Klemm, Andreas Nowak

**Affiliations:** ^1^ Department of Otorhinolaryngology, Head and Neck Surgery, Plastic Surgery, Städtisches Klinikum Dresden, Dresden, Germany; ^2^ Department of Anesthesiology and Intensive Care Medicine, Emergency Medicine and Pain Management, Städtisches Klinikum Dresden, Dresden, Germany

**Keywords:** tracheostomy, tracheal ring fracture, histology, tracheal stenosis, intensive care unit

## Introduction

In this issue of Pathology & Oncology Research, Bódis et al. compare incidences of cartilage injury caused by different percutaneous dilatation techniques, including Single Dilator, Griggs’ and modified bidirectional Griggs’ method in a randomized cadaver study. Based on the data reported, the authors conclude that both standard and modified Griggs’ forceps dilatational methods were safer than Single Dilator in respect of cartilage injury [1]. Pathological studies of the trachea after a tracheostomy are important because technical aspects also play a role in the decision to perform a tracheostomy. In our opinion an important aspect is the fact that tracheal rings in critical care patients are not homogeneously composed of hyaline cartilage.

## Histological analysis

A histological analysis was performed on the midsections of the second and third tracheal rings of 103 mechanically ventilated adult patients who were critically ill and had undergone open surgical tracheostomy. Of these cases observed, only 26 (25%) had uniform hyaline cartilage structure. Signs of physiologic aging and reactive changes due to previous microtrauma and severe underlying disease were present in all other cases. The changes concerned compact and cancellous bone structures in 26%, partly with orthologous hematopoietic marrow. Dystrophic degenerative cartilage calcifications were observed in 20% of cases, while 13% had proliferation of cartilaginous tissue with brood capsules. Dystrophic to non-viable cartilage zones were reported in 4% of cases, while 3% had osseous metaplasia in connective tissue and 2% had cartilage and mucosal necrosis. Inflammatory reactions in mucosal and connective tissue areas were observed in 32% of cases. No correlations between age and different histological tissue qualities or reactive changes were found [2].

## Discussion

Studies have recognized that the histologic structure can explain tracheal ring fractures in percutaneous dilated tracheotomies (PDT). A systematic search detected fractures of tracheal rings in 17% of PDTs [3]. Fractures of the tracheal rings might initiate the development of tracheal stenosis. The literature reports a risk of 1%–5% for the development of tracheal stenosis due to additional inflammation, depending on the method of PDT and the definition of the stenosis. If the trachea is involved in inflammatory processes, open surgical tracheotomy should be preferred, since tracheal ring fractures in open surgical tracheotomy are rare (0.9%) [3]. The current knowledge on alteration and inflammation of tracheal tissue in critically ill patients during mechanical ventilation exhibits an overall low quality of evidence. Bódis et al. suggest that with good visibility due to surgical preparation of pre-tracheal structures, endoscopic control during PDT is nearly unnecessary [1]. It is our recommendation that endoscopic control and resection of dislocated tracheal ring fragments should always be performed after PDT because of the risk of dislocated tracheal ring fracture with subsequent tracheal stenosis. Among the numerous worldwide tracheostomies performed, the focus should be on the reasons for the development of tracheal stenosis after tracheostomy, their risk factors and methods to avoid them.

## References

[1] Bódis F Orosz G Tóth JT Szabó M Élő LG Gál J Percutaneous tracheostomy: comparison of three different methods with respect to tracheal cartilage injury in cadavers-randomized controlled study. Pathol Oncol Res (2023) 29:1610934. 10.3389/pore.2023.1610934 37123534 PMC10135429

[2] Klemm E Nowak A Haroske G. Histomorphologische Befunde der 2. und 3. Trachealspange von Tracheotomiepatienten der Intensivmedizin. GMS Curr Posters Otorhinolaryngol Head Neck Surg (2011) 7:Doc43 10.3205/cpo000632

[3] Nowak A Kern P Koscielny S Usichenko T Hahnenkamp K Feasibility and safety of dilatational tracheotomy using the rigid endoscope: a multicenter study. BMC Anesthesiol (2017) 17:7. 10.1186/s12871-017-0301-y 28088174 PMC5237481

